# Anterior variable angle locking neutralisation plate superiority over traditional tension band wiring for treating transverse patella fractures

**DOI:** 10.1002/jeo2.12088

**Published:** 2024-07-05

**Authors:** Majd Tarabichi, Nirav Mungalpara, Asher Lichtig, Sunjung Kim, Joseph Karam, Jason Koh, Farid Amirouche

**Affiliations:** ^1^ Department of Orthopaedic Surgery University of California, San Francisco San Francisco California USA; ^2^ Department of Orthopaedic Surgery University of Illinois Chicago Chicago Illinois USA; ^3^ Department of Orthopaedic Surgery Northshore University HealthSystem Skokie Illinois USA; ^4^ Institute of Orthopaedics and Spine Northshore University HealthSystem Skokie Illinois USA

**Keywords:** anterior variable angle locking neutralisation plate, cannulated screws, cyclic loading test, tensile failure, tension band wire, transverse patella fracture

## Abstract

**Purpose:**

This paper investigates the biomechanical benefits of using hybrid constructs that combine cannulated screws with tension band wiring (TBW) cerclage compared to cannulated screws with anterior Variable Angle locking neutralisation plates (VA LNP). These enhancements can bear heavier loads and maintain the repaired patella's integrity, in contrast to traditional methods.

**Method:**

Eighteen fresh‐frozen human cadaver patellae were carefully fractured transversely at their midpoints using a saw. They were then divided into two groups of nine for subsequent utilisation. Fixation methods included Cannulated Screw Fixation added with either TBW or VA LNP Fixation Technique. Cyclic loading simulations (500 cycles) were conducted to mimic knee motion, tracking fracture displacement with Optotrak. After that, the constructs were secured over a servo‐hydraulic testing machine to determine the load‐to‐failure on axial mode.

**Results:**

The average fracture displacement for the anterior neutralisation plate group was 0.09 ± 0.12 mm, compared to 0.77 ± 0.54 mm for the tension band wiring with cannulated screw group after 500 cyclic loading. This result is statistically significant (*p* = 0.004). The anterior neutralisation plate group exhibited a mean load‐to‐failure of 1359 ±21.53 N, whereas the tension band wiring group showed 780.1 ± 22.62 N, resulting in a significant difference between the groups (*p* = 0.007).

**Conclusion:**

This research highlights the superior biomechanical advantage of VA LNP over TBW for treating simple transverse patella fractures with two cannulated screws. It also highlights how the TBW is still a valuable option considering the load‐to‐failure limit.

**Level of Evidence:**

Not Applicable.

**Abbreviations**
AO: arbeitsgemeinschaft für osteosynthesefragen
K wire: kirschner wires
KOS ADLS: Knee Outcome Survey Activities of Daily Living Scale
RTA: road traffic accidents
TBW: tension band wiring
VA LNP: variable angle locking neutralisation plates

## BACKGROUND

Patella functions as a mechanical pulley during knee extension, augmenting the extensor forces by up to 30% [16]. This biomechanical advantage is particularly noticeable in the final 30 degrees of extension [11]. Patella fractures constitute 0.5%–1.5% of all skeletal injuries [29]. The most common cause is road traffic accidents (RTA), followed by sports‐related injuries [2]. These fractures may result from direct or indirect forces applied to the patella through excessive quadriceps contraction. The transverse patella fracture is the most prevalent among all kinds of patella fractures [12].

The most common fixation method for transverse patella fractures includes Kirschner wires (K‐wires) or parallel cannulated screws, with tension band cerclage wiring (TBW) in a figure‐eight configuration [4, 5, 9]. Conventional constructs, such as K‐wires, screws, and cerclage wiring, used for fracture fixation, have limited biomechanical strength.

However, it has been observed that suboptimal implant quality or some anatomical factors can compromise outcomes. An additional augmentation is often necessary, such as in elderly osteoporotic patients, who require more healing time [14, 22]. Augmenting routine constructs is a feasible solution to enhance stability in such instances. There is a lack of literature on the biomechanical analysis of such hybrid augmented constructs for simple transverse patella fractures.

This study focuses on the comparative biomechanical analysis of two hybrid constructs for transverse patella fracture fixation. We compare combined cannulated screws with tension band cerclage wiring (TBW) and cannulated screws with an anterior variable angle locking neutralisation plate (VA LNP), both following the fracture fixation principles of Arbeitsgemeinschaft für Osteosynthesefragen (AO). We hypothesise that the hybrid construct, consisting of cannulated screws combined with an anterior VA LNP, will demonstrate superior load‐bearing capacity and improved fracture reduction retention during cyclic loading compared to traditional constructs such as cannulated screws with TBWs.

## MATERIALS AND METHODS

### Cadaveric sample preparation

Eighteen fresh frozen human cadaver patellae, devoid of any knee injury or surgery history, were utilised in this study. The average age was 78 years, with a range of 46–98 years. The average weight was 151 lbs (109–193 Range). The patellae were evenly distributed into two groups, each comprising nine specimens. Transverse fractures were induced in all patellae at the midline using a sagittal saw. We excluded specimens that exhibited any signs of chondromalacia or osteochondral defects, had congenital morphological anomalies of the patella, or showed any evidence of arthritis or peripheral osteophytes.

### Cannulated screw fixation

After creating transverse fractures, each patella underwent fixation with cannulated screws. Large, pointed reduction forceps were employed to achieve reduction. Preliminary reduction was maintained using two 1.25‐mm K‐wires while assessing optimal screw positioning. Subsequently, a cannulated 2.7‐mm drill was used for screw canal preparation. Stainless steel partially threaded 3.5‐mm cannulated screws (DePuy Synthes), ranging from 24 to 34 mm long, were meticulously inserted. At this juncture, the surgical protocol diverged between the two groups.

### Tension band wiring technique

**Figure 1:**
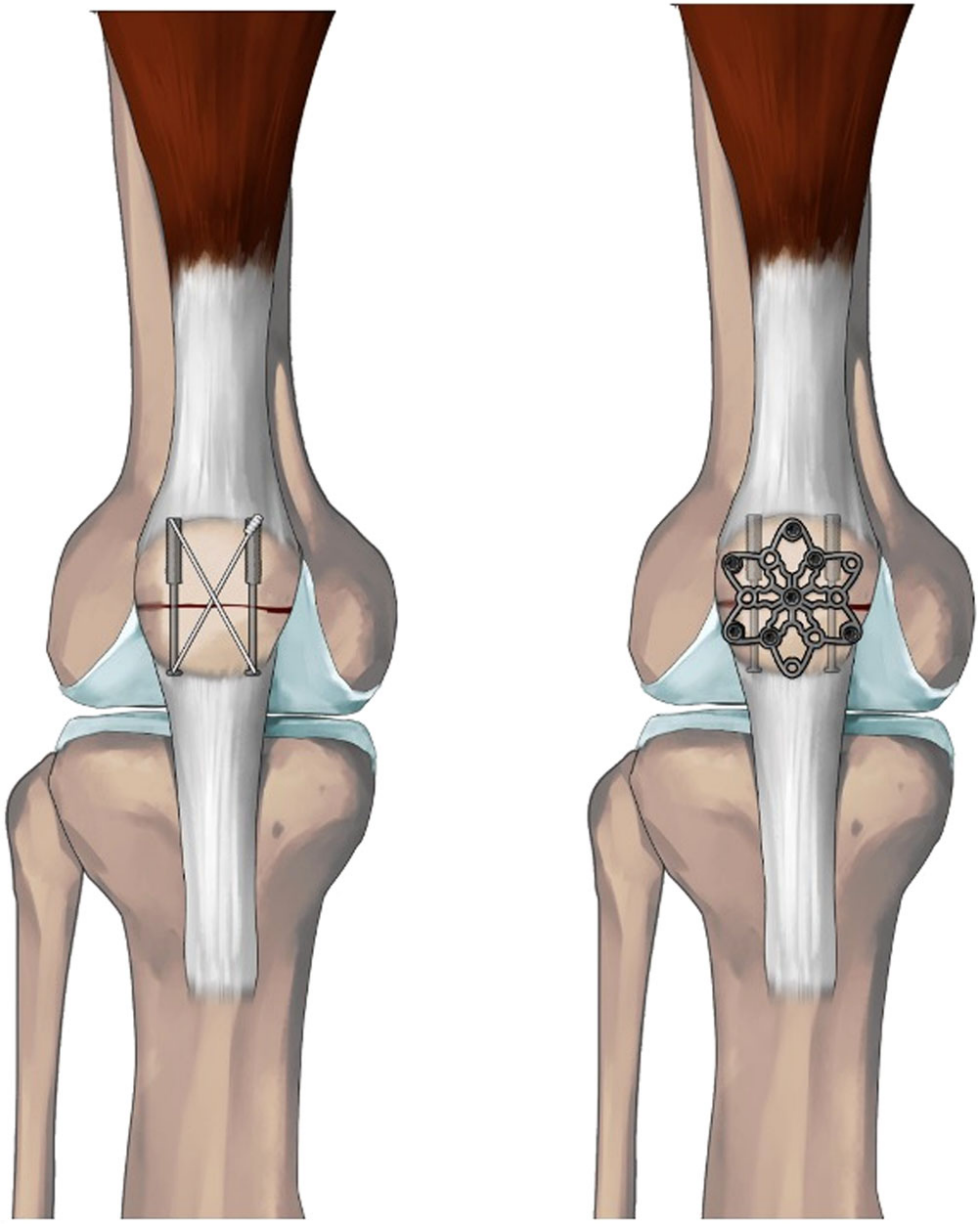
The final constructs consist of (a) cannulated screws and tension band wiring and (b) cannulated screws and variable angle locking neutralization plates.

For the tension band wiring group, a 16G stainless steel wire (DePuy Synthes) was inserted through the 3.5 mm cannulated screws, traversing the anterior aspect of the patella. The soft tissues were removed from the wires, followed by symmetrical tightening through twisting with pliers (Figure 1).

### Plate fixation technique

An appropriately sized stainless steel 2.7 mm Anterior VA LNP (DePuy Synthes) was selected in the plate group. The plate was contoured using specialised plate benders to achieve a flush fit against the anterior cortex of the patella. Seven 2.7‐mm locking screws were positioned in proximal and distal segments (Figure 1).

### Cyclic loading test

The experimental procedure involved the utilisation of a customised testing rig to mount the patellae. The cyclic test was conducted using a customised rig and LabVIEW software (National Instruments™). The rig was constructed to mimic the knee joint's full extension and subsequent 90° flexion. To simulate the patella's motion, each sample was fastened to the rig using multiple loops of string attached to the quadriceps and the base of the patellar ligament. The string was then affixed to a servo‐hydraulic shaft, facilitating the movement of the pate and achieving the desired angle. Aluminium wires were attached to the end of the patellar ligament to impart a cyclic load onto the samples. These wires supported two bars with a combined weight of 1 kg, simulating the load exerted by the lower leg and foot while standing. The total weight, including aluminium support, amounted to 3.1 kg, effectively replicating the weight of the foot and leg, as per the earlier published studies [5, 8, 27].

**Figure 2:**
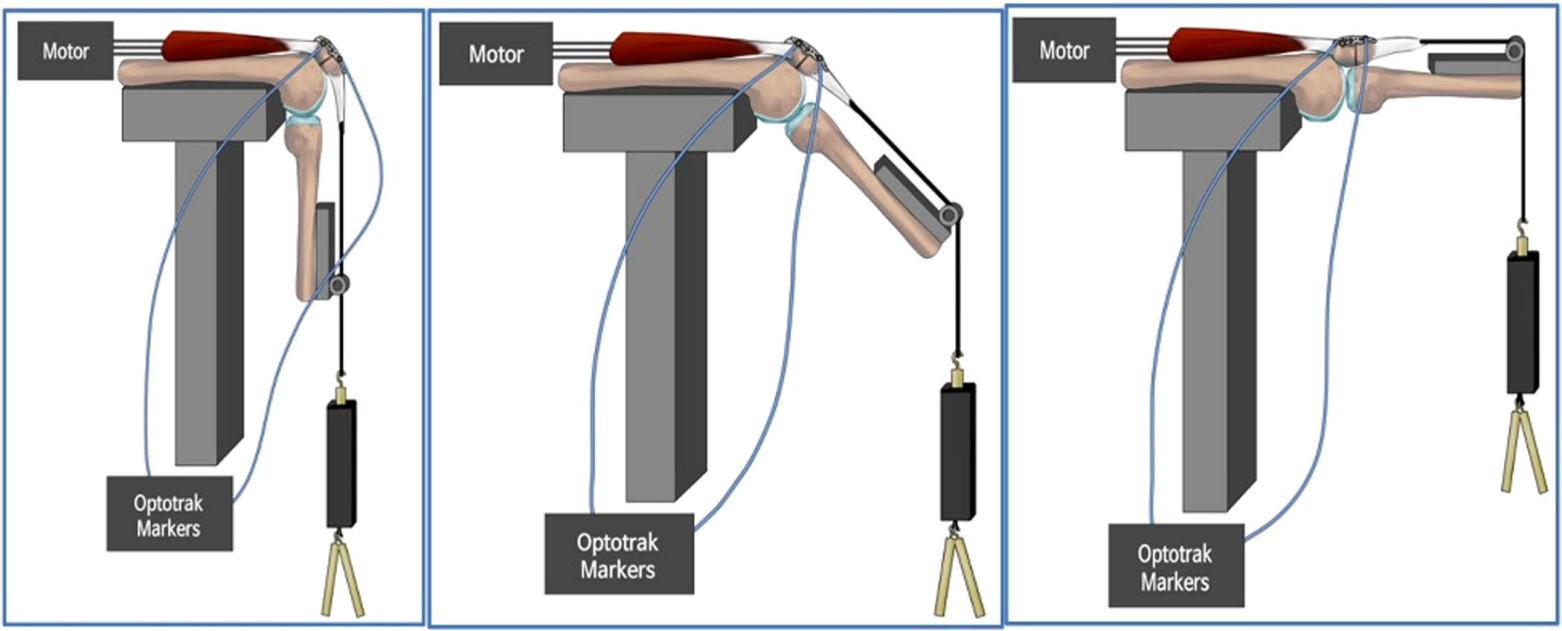
Loaded patella in a 90‐degree flexed position (top left), 45‐degree flexion (centre), and to the complete extension (top right). This cycling apparatus with optical tackers is labelled using Optotrak to determine fracture displacement.

The specimen was subjected to 500 cycles of loading, each cycle encompassing an 8‐s loading phase and an 8‐s unloading phase, ranging from 0 to 150 N (with a frequency of 0.12 Hz). Prior research has corroborated that this specific loading protocol accurately simulates the knee moment experienced by an average individual weighing 70 kg (Equivalent to our average weight) [5, 23]. During the experiment, displacement and force measurements were captured utilising a digital data acquisition system, as illustrated in Figure 2. The details of the testing apparatus included a motor operating at a speed of 1/16 cycles per second and a travel distance of about 25 cm. This travel distance was equivalent to an approximately 90‐degree rotational angle, achieved with the testing apparatus having a radius of 6.5 inches. To precisely gauge the distance between the patella fragments before and after testing, a sophisticated 3D optical motion capture system, NDI Optotrak (Optotrak Certus; NDI), was employed with an accuracy of 0.1 mm. This system utilised 7‐mm diameter IRED (Infralight‐emitting Diode) encased markers. These two markers, strategically positioned above and below the partial cut on the patella, were interconnected to the NDI system control unit via the marker strobe. A position sensor, adept at capturing the emitted infrared light, was aligned facing the markers. This setup facilitated the precise recording of marker displacement at various junctures during the cyclic loading, with each sensor capable of discerning its location in three‐dimensional space (*x‐*, *y‐*, and *z*‐axis). The cumulative effect of this sophisticated setup was the successful documentation of alterations in the fracture gap throughout the cyclic loading.

### Load‐to‐failure protocol

**Figure 3:**
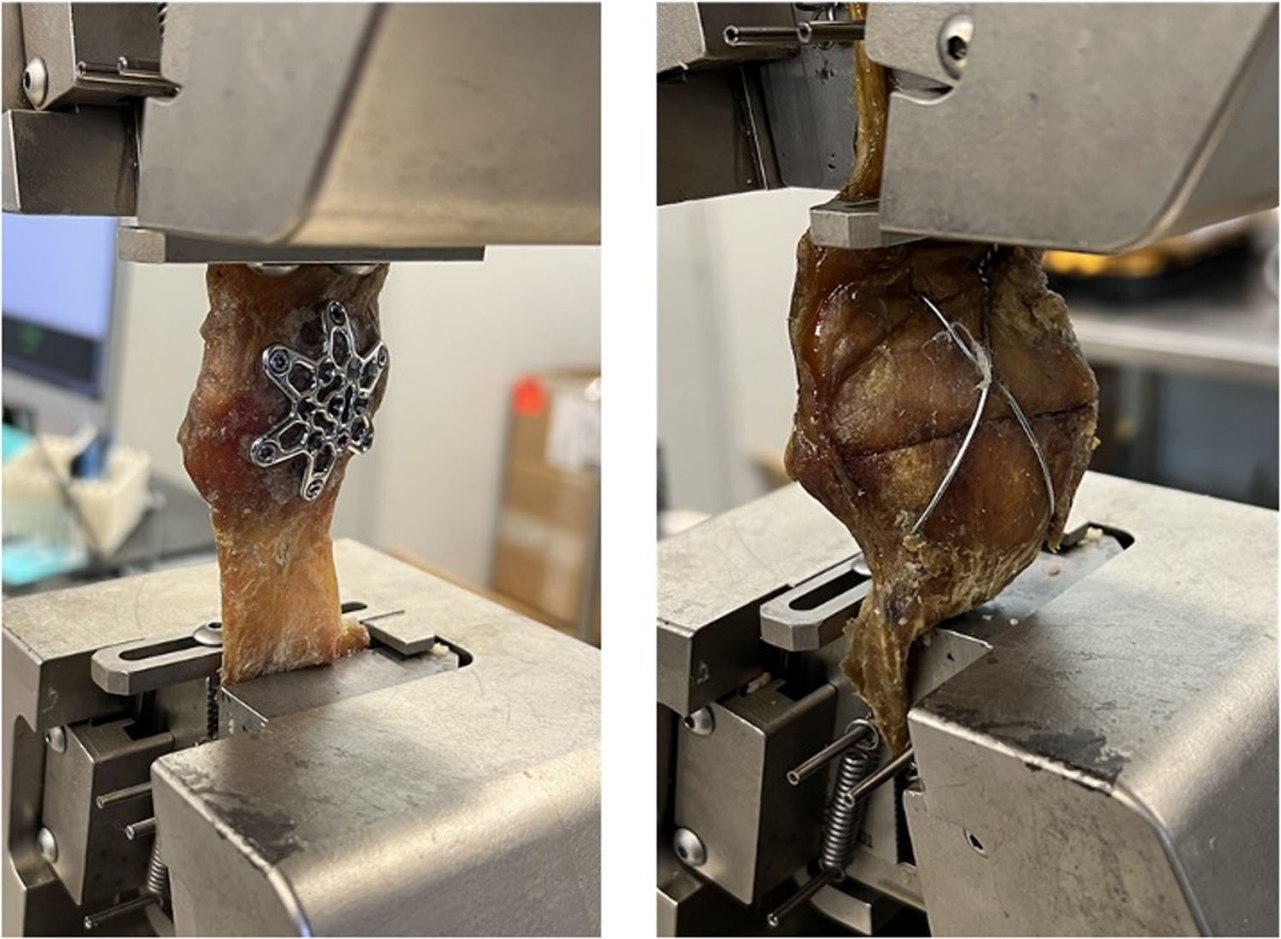
The MTS jaw fixture features a construct of the plate and cannulated screws (left) and a construct of tension band wiring and cannulated screws (right).

The load‐to‐failure test for each patella was performed after the cyclic loading test (Figure 3). A servohydraulic testing machine (Model C44, Criterion Electromechanical Test System by MTS Systems Corporation, Eden Prairie, MN, USA) was used, with tensile gripping clamps securing the top and bottom of the specimen. The quadriceps tendon and patellar ligament were firmly fastened into the machine to eliminate the risk of slippage. The grip enclosed approximately two‐thirds of the quadriceps tendon and patella ligament to ensure an even load distribution during the test. The testing machine was equipped with a sensor that automatically detected the load and terminated the test at the point of failure, which was defined as a fracture gap of 2 mm or more. The tensile sensor's pulling speed was 1 mm per second, and the machine's data acquisition rate was 10 Hz. Figure 3 highlights how specimens were positioned in the MTS machine. The load was increased progressively until the specimen reached its failure point. A digital displacement sensor was used to record the displacement, while a load cell was employed to record the load. Before conducting the tensile tests, each tendon's width, height, and thickness were measured to ascertain their dimensions. The tests were conducted in a controlled environment to mitigate potential external factors that could have influenced the results. To uphold the accuracy and reliability of the results, all specimens were subjected to uniform testing conditions, including identical load rates and grip positions.

### Data analysis

In the experimental procedure, each patella underwent 500 extension cycles. The positions of markers on the patella were recorded at 0.01‐s intervals during these cycles. The gap between the two markers was calculated using their 3D coordinates at each time point, quantifying the gap opening during cycling. For ease of subsequent data analysis, the cycles were grouped in batches of 100, with the maximum and minimum distances within each batch identified. A single gap measurement was then derived from these extreme values. As a result, five gap values were recorded for each patella, allowing for observation of the gap's evolution throughout the cycling process. Tensile testing was also performed on each patella sample using the MTS machine, with the failure force determined from the force‐displacement curve.

### Statistical analysis

The Shapiro–Wilk test was utilised to evaluate the normality of data distribution within each group. This was followed by implementing the Kruskal–Wallis test to gauge the significance of the differences observed across multiple groups. Post hoc analysis employed the Mann–Whitney *U* test to identify specific differences between groups and determine any statistically significant variations.

## RESULTS

**Figure 4:**
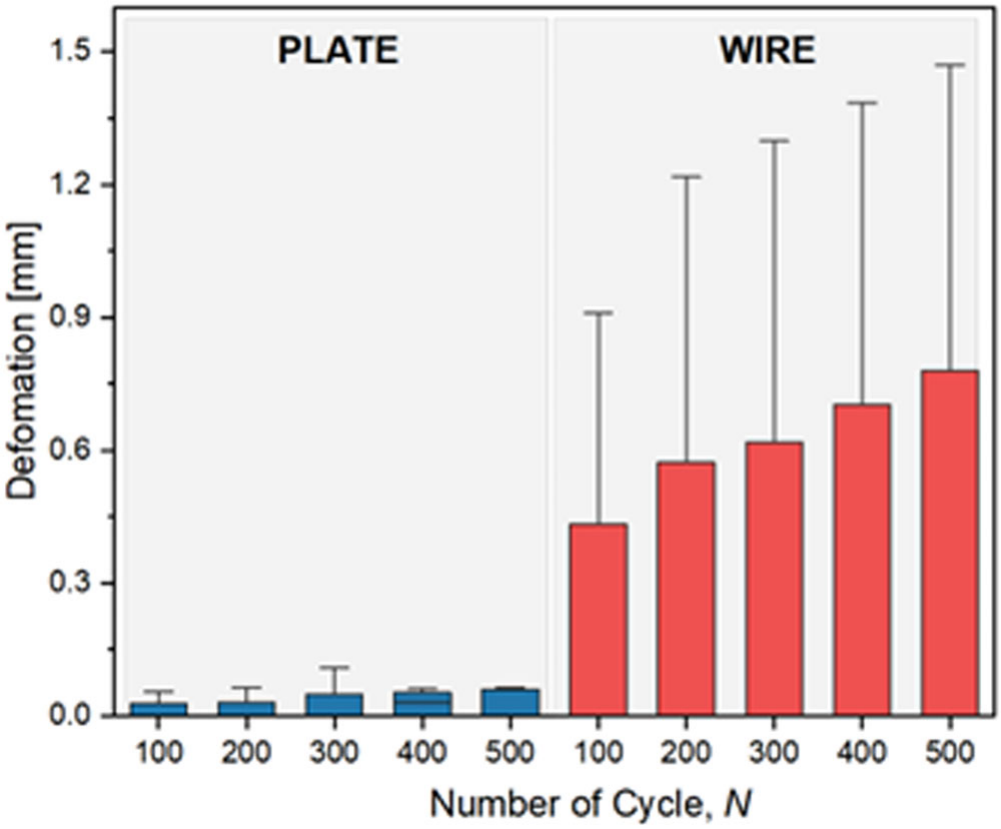
Compares average deformation values between the plate and wire fixation methods across cycles 100–500. Plate fixation consistently exhibited lower deformation, with an average of 0.060 mm at cycle 100, gradually increasing to 0.099 mm at cycle 500. In contrast, wire fixation displayed higher deformation, starting at an average of 0.433 mm at cycle 100 and rising to 0.778 mm at cycle 500.

**Figure 5:**
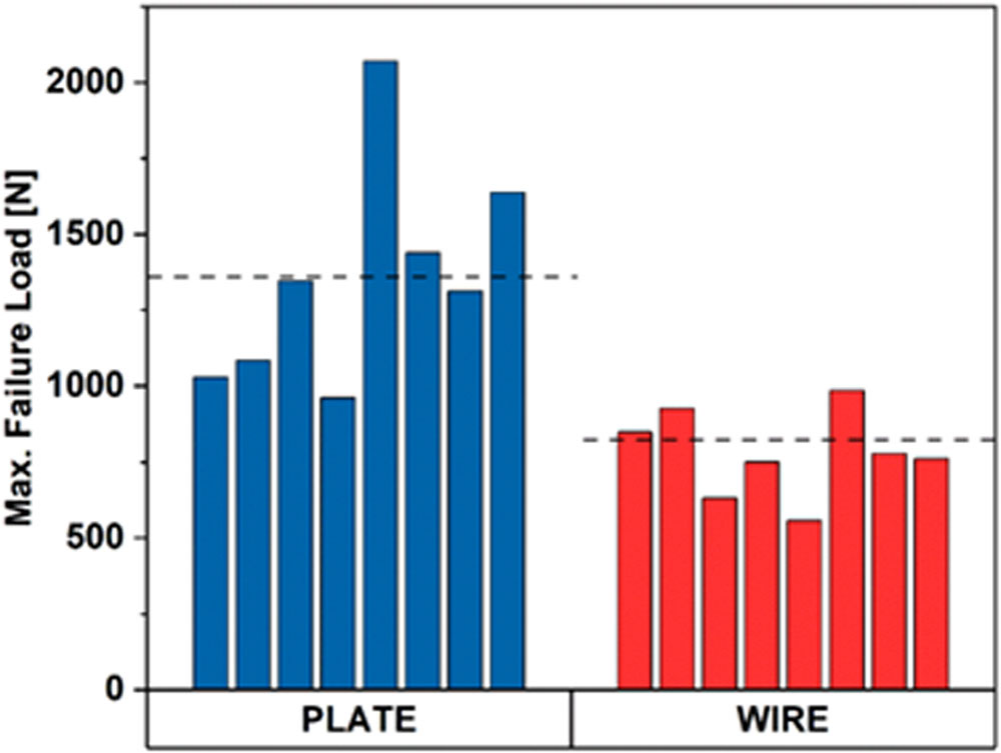
Maximum failure force of plate and wire fixation systems during tensile testing. The graph shows the maximum failure force for plate and wire fixation systems during tensile testing. Plate fixation demonstrated a higher average maximum failure force than wire fixation, with plate fixation exhibiting an average maximum failure force of 1359 N, while wire fixation had a comparatively lower average maximum failure force of 780.1 N.

Two specimens, precisely one employing plate construction and another utilising cannulated screw with tension band wiring construction, experienced catastrophic failure below the 400 N threshold, presumably due to compromised bone quality. Consequently, these instances were excluded from the study. Table 1, as well as Figures 4 and 5, illustrates the data of our experiment.

**Table 1:** Data of the fracture site displacement and load‐to‐failure.

Sample no	Fixation method	Cyclic testing for deformation (fracture site displacement) (mm)						Tensile testing (load‐to‐failure)
	PLATE/WIRE	0	100 cycles	200 cycles	300 cycles	400 cycles	500 cycles	Peak force (*N*)
1	PLATE	0	0.291	0.356	0.376	0.38	0.369	1029
2	PLATE	0	0.054	0.064	0.108	0.166	0.208	1083
3	PLATE	0	0.016	0.014	0.06	0.02	0.029	1346
4	PLATE	0	0.001	0.003	0.002	0.017	0.02	963
5	PLATE	0	0.018	0.01	0.015	0.019	0.02	2069
6	PLATE	0	0.024	0.025	0.028	0.03	0.031	1438
7	PLATE	0	0.03	0.042	0.068	0.061	0.063	1312
8	PLATE	0	0.046	0.05	0.051	0.055	0.051	1637
	Avg	0	0.060	0.071	0.089	0.094	0.099	1359.625
1	WIRE	0	0.178	0.222	0.242	0.236	0.234	849
2	WIRE	0	0.498	0.798	0.872	0.989	1.106	927
3	WIRE	0	0.909	1.217	1.298	1.384	1.469	631
4	WIRE	0	0.72	0.896	0.974	1.129	1.249	751
5	WIRE	0	0.732	0.899	0.877	1.128	1.249	558
6	WIRE	0	0.082	0.089	0.173	0.187	0.217	986
7	WIRE	0	0.062	0.105	0.126	0.14	0.157	778
8	WIRE	0	0.285	0.353	0.391	0.418	0.544	761
	Avg	0	0.433	0.572	0.619	0.701	0.778	780.125

### Fracture site displacement

The displacement at the fracture site was recorded at different cycles—1, 100, 200, 400 and 500—on the custom testing apparatus while subjecting both groups to a 10 N load (Figure 4). A biomechanical analysis for the TBW and plate construction is detailed in Table 1. Post 500 cycles, the average fracture displacement was observed to be 0.09 ± 0.12 mm for the plate construction and 0.77 ± 0.54 mm for the cannulated screw and wire construction. A two‐tailed *T*‐test for independent means established a significant disparity between the two groups (*p* = 0.004).

### Load‐to‐failure analysis

The load‐to‐failure outcomes, alongside the comprehensive results, are outlined in Figure 5. The mean load‐to‐failure for the anterior VA LNP and cannulated screw construction was 1359 N (SD = 393.30), while for the cannulated screw with tension band wiring construction was 780.1 N (SD = 233). A two‐tailed T‐test for independent means indicated a statistically significant difference between the groups (*p* = 0.007). Figure 5 is the graph illustrating our experimental data.

## DISCUSSION

The conventional method to treat simple transverse patella fractures typically involves K wires with tension band wiring (TBW) or cannulated screws. Berg et al. [3] showed clinical advantages of using cannulated screws over K wires. Numerous studies highlighted the prevalence of symptomatic implants and the frequent need for their removal, especially in cases using traditional TBW instead of cannulated screws [6, 28]. Kumar et al. [18] reported an implant removal rate of 40% in patients under 60 years of age treated with TBW and K wires. Similarly, Hoshino et al. [10]. observed implant removal rates of 37%, in conventional K‐wire fixation compared to 23% in cannulated screw fixation. Some studies have indicated that patients with K‐wires experience twice the incidence of symptomatic implants compared to those with cannulated screws [10]. Carpenter et al. [5] demonstrated that TBW with two cannulated screws outperforms K wires and a TBW construct or a single screw alone regarding load to failure, using cadaver models. Additionally, Thelen et al. [27], through repetitive cycling on polyurethane foam patellae, showed that cannulated screws with TBW are superior to conventional K wires with TBW in reducing fracture gaps.

However, for certain groups, such as elderly patients and those with severe osteoporosis [14], it is necessary to reconsider traditional fixation constructs to improve strength and stability. Additionally, some patients might require more flexibility in their post‐op immobilisation. This necessitates an augmentation beyond the standard fixation methods. Augmentation utilising cannulated screws can be achieved through either TBW or an Anterior VA LNP. Our study provides a comparative biomechanical analysis, comparing two constructs: cannulated screws with TBW versus cannulated screws supplemented by an Anterior VA LNP.

Anterior plating has emerged as a superior construct for patella fractures, offering enhanced biomechanical support compared to TBW. Fixed‐angle plating, as demonstrated by Thelen et al. [27] reduces displacement and increases failure loads compared to cannulated lag screws with TBW. The recently developed anterior VA LNP offers increased flexibility in screw placement, accommodating screws of various sizes. These plates are precontoured based on patella morphology, resulting in decreased interfragmentary displacement compared to tension band wiring, as reported by Stoffel et al. [26]. They analysed TBW with cannulated screws versus VA LNP on simple two‐part fractures and complex five‐part patella fractures using cadaver models. This study reported significantly smaller displacement following cycling in the plate construction compared to the TBW construction. Similar results were reported by Kfuri et al. [17]. Moreover, low‐profile plates contribute to reduced anterior knee pain and superior outcomes, such as better Knee Outcome Survey Activities of Daily Living Scale (KOS‐ADLS) scores, surpassing tension band constructs [19, 30]. The biomechanical advantage of VA LNP in patella fracture has been repeatedly demonstrated [1, 7, 15, 17]. However, little data has been published on the effectiveness of anterior VA LNP versus TBW as an augmentation in simple transverse fractures of the patella fixed with two cannulated screws.

Our study reveals that the anterior VA LNP and the TBW construct demonstrate displacement rates within acceptable limits for primary healing. However, after 500 cycles, a notable disparity emerges between the constructions: the anterior VA LNP shows an average displacement of 0.09 ± 0.12 mm, whereas the TBW construct demonstrates a 0.77 ± 0.54 mm displacement. Despite this discrepancy, as established by previous studies, both groups exceed the minimal threshold for direct healing (0.01 mm) and fall below the upper limit for indirect healing (0.8–1.0 mm) [13, 17, 20, 24]. While the statistical superiority of the plate construct is evident, the clinical significance of this difference may be marginal and not entirely justify the associated increased cost.

Shelburne et al. [25] utilising mathematical model, estimated maximal loads on knee joint through muscles and ligaments. They find that the peak force on the patella tendon only approximates 300 N, which falls below the minimum values observed in either group of our study. This shows the substantial load‐bearing capacity of the anterior VA LNP construction (1267.20 ± 393.30 N) and the TBW construction (820.00 ± 233.00 N), further supporting our findings. More stable and sturdy fixation by both constructs may allow earlier active quadriceps range of motion. Early mobilisation can reduce rates of quadriceps atrophy and extensor mechanism weakness [21]. However, aggressive postoperative protocols increase the risk of failure; the augmentation method may also mitigate this risk.

This study is limited due to the use of in vitro examination of these two fixation and augmentation techniques and, therefore, does not consider clinical aspects such as biological healing response, soft tissue contributions to stability, and individual patient factors. Additionally, this study included two samples that underwent catastrophic failure below 400 N, bringing into question the tissue quality as no measurements of bone mineral density were taken. Finally, the comparison of this study is limited due to the varied methodology presented in the literature examining the biomechanics of patella fracture fixation methods. Another limitation of our study was the use of isolated patellae, and testing was carried out by placing each patella over standard sawbones and a customised rig. Without the environment of a knee joint, the patella likely did not reflect the exact physiologic condition.

## CONCLUSION

This research highlights the superior biomechanical advantage of anterior VA LNP over TBW for treating simple transverse patella fractures with two cannulated screws. However, TBW was still a valuable approach, given that its load to failure is above the acceptable threshold. Further rigorous randomised trials are essential to validate these findings, assess clinical viability, and consider financial implications. Such augmentations are particularly crucial for geriatric patients and those with severe osteoporosis or in those situations where the quality of implants can be unpredictable.

## AUTHOR CONTRIBUTIONS

Under the guidance of the three senior authors, Farid Amirouche, Jason Koh and Joseph Karam, Majd Tarabichi conceptualised and designed the experimental framework, Asher Lichtig and Sunjung Kim conducted the experiments and carried out the data analysis. Nirav Mungalpara was responsible for composing the manuscript.

## CONFLICT OF INTEREST STATEMENT

The authors declare no conflict of interest.

## ETHICS STATEMENT

Cadaver experiments were performed in a biomechanics laboratory of our institution in accordance with the regulations of the Institution's Ethics Committee. Obtaining ethical approval from this committee was waived due to the ex vivo cadaver nature of this study.

## Data Availability

The datasets used and/or analysed during the current study are available from the corresponding author upon reasonable request.

## References

[1] Banks, K.E. , Ambrose, C.G. , Wheeless, J.S. , Tissue, C.M. & Sen, M. (2013) An alternative patellar fracture fixation: a biomechanical study. Journal of Orthopaedic Trauma, 27(6), 345–351. Available from: 10.1097/BOT.0b013e31826623eb 22773018

[2] Benli, I.T. , Akalin, S. , Mumcu, E.F. , Citak, M. , Kiliç, M. & Paşaoğlu, E. (1992) The computed tomographic evaluation of patellofemoral joint in patellar fractures treated with open reduction and internal fixation. The Kobe Journal of Medical Sciences, 38(4), 233–243. 1469888

[3] Berg, E.E. (1997) Open reduction internal fixation of displaced transverse patella fractures with figure‐eight wiring through parallel cannulated compression screws. Journal of Orthopaedic Trauma, 11(8), 573–576. Available from: 10.1097/00005131-199711000-00005 9415863

[4] Burvant, J.G. , Thomas, K.A. , Alexander, R. & Harris, M.B. (1994) Evaluation of methods of internal fixation of transverse patella fractures: a biomechanical study. Journal of Orthopaedic Trauma, 8(2), 147–153. Available from: 10.1097/00005131-199404000-00012 8207572

[5] Carpenter, J.E. , Kasman, R.A. , Patel, N. , Lee, M.L. & Goldstein, S.A. (1997) Biomechanical evaluation of current patella fracture fixation techniques. Journal of Orthopaedic Trauma, 11(5), 351–356. Available from: 10.1097/00005131-199707000-00009 9294799

[6] Catalano, J.B. , Iannacone, W.M. , Marczyk, S. , Dalsey, R.M. , Deutsch, L.S. , Born, C.T. et al. (1995) Open fractures of the patella: long‐term functional outcome. The Journal of Trauma: Injury, Infection, and Critical Care, 39(3), 439–444. Available from: 10.1097/00005373-199509000-00007 7473905

[7] Dickens, A.J. , Salas, C. , Rise, L. , Murray‐Krezan, C. , Taha, M.R. , DeCoster, T.A. et al. (2015) Titanium mesh as a low‐profile alternative for tension‐band augmentation in patella fracture fixation: a biomechanical study. Injury, 46(6), 1001–1006. Available from: 10.1016/j.injury.2015.02.017 25769202

[8] Domby, B. , Henderson, E. , Nayak, A. , Erdoğan, M. , Gutierrez, S. , Santoni, B.G. et al. (2012) Comparison of cannulated screw with tension band wiring versus compressive cannulated locking bolt and nut device (CompresSURE) in patella fractures ‐ a cadaveric biomechanical study. Journal of Orthopaedic Trauma, 26(12), 678–683. Available from: 10.1097/BOT.0b013e31826f5985 22932750

[9] Gwinner, C. , Märdian, S. , Schwabe, P. , Schaser, K.‐D. , Krapohl, B.D. & Jung, T.M. (2016) Current concepts review: fractures of the patella. GMS Interdisciplinary Plastic and Reconstructive Surgery DGPW, 5, Doc01. 10.3205/iprs000080 26816667 PMC4717300

[10] Hoshino, C.M. , Tran, W. , Tiberi, J.V. , Black, M.H. , Li, B.H. , Gold, S.M. et al. (2013) Complications following tension‐band fixation of patellar fractures with cannulated screws compared with Kirschner wires. Journal of Bone and Joint Surgery, 95(7), 653–659. Available from: 10.2106/JBJS.K.01549 23553301

[11] Huberti, H.H. & Hayes, W.C. (1984) Patellofemoral contact pressures. The influence of q‐angle and tendofemoral contact. The Journal of Bone & Joint Surgery, 66(5), 715–724. Available from: 10.2106/00004623-198466050-00010 6725318

[12] Jarraya, M. , Diaz, L.E. , Arndt, W.F. , Roemer, F.W. & Guermazi, A. (2016) Imaging of patellar fractures. Insights into Imaging, 8(1), 49–57. Available from: 10.1007/s13244-016-0535-0 27905071 PMC5265199

[13] Kaderly, R.E. (1991) Primary bone healing. Seminars in Veterinary Medicine and Surgery (Small Animal), 6(1), 21–25. 2038620

[14] Kammerlander, C. , Neuerburg, C. , Verlaan, J.‐J. , Schmoelz, W. , Miclau, T. & Larsson, S. (2016) The use of augmentation techniques in osteoporotic fracture fixation. Injury, 47(Suppl 2), S36–S43. Available from: 10.1016/S0020-1383(16)47007-5 27338226

[15] Karakasli, A. , Acar, N. , Ertem, F. , Ozmanevra, R. & Erduran, M. (2017) A novel anatomical patellar plate for transverse patellar fracture: a biomechanical in‐vitro study. Acta Orthopaedica et Traumatologica Turcica, 51(4), 337–341. Available from: 10.1016/j.aott.2017.04.006 28554845 PMC6197350

[16] Kaufer, H. (1971) Mechanical function of the patella. The Journal of Bone & Joint Surgery, 53(8), 1551–1560. Available from: 10.2106/00004623-197153080-00007 5121795

[17] Kfuri, M. , Escalante, I. , Schopper, C. , Zderic, I. , Stoffel, K. , Sommer, C. et al. (2021) Comminuted patellar fractures: the role of biplanar fixed angle plate constructs. Journal of Orthopaedic Translation, 27, 17–24. Available from: 10.1016/j.jot.2020.10.003 33344168 PMC7732873

[18] Kumar, G. , Mereddy, P.K. , Hakkalamani, S. & Donnachie, N.J. (2010) Implant removal following surgical stabilization of patella fracture. Orthopedics, 33(5), 301–304. Available from: 10.3928/01477447-20100329-14 20506948

[19] Lorich, D.G. , Fabricant, P.D. , Sauro, G. , Lazaro, L.E. , Thacher, R.R. , Garner, M.R. et al. (2017) Superior outcomes after operative fixation of patella fractures using a novel plating technique: a prospective cohort study. Journal of Orthopaedic Trauma, 31(5), 241–247. Available from: 10.1097/BOT.0000000000000787 28166170

[20] Marsell, R. & Einhorn, T.A. (2011) The biology of fracture healing. Injury, 42(6), 551–555. Available from: 10.1016/j.injury.2011.03.031 21489527 PMC3105171

[21] Mohtadi, N. (2005) Injured limbs recover better with early mobilization and functional bracing than with cast immobilization. The Journal of Bone & Joint Surgery, 87(5), 1167. Available from: 10.2106/00004623-200505000-00042 15866989

[22] Rommens, P.M. (2019) Paradigm shift in geriatric fracture treatment. European Journal of Trauma and Emergency Surgery, 45(2), 181–189. Available from: 10.1007/s00068-019-01080-x 30725152

[23] Schnabel, B. , Scharf, M. , Schwieger, K. , Windolf, M. , Pol, B. , Braunstein, V. et al. (2009) Biomechanical comparison of a new staple technique with tension band wiring for transverse patella fractures. Clinical Biomechanics, 24(10), 855–859. Available from: 10.1016/j.clinbiomech.2009.08.002 19716216

[24] Shapiro, F. (1988) Cortical bone repair. The relationship of the lacunar‐canalicular system and intercellular gap junctions to the repair process. The Journal of Bone & Joint Surgery, 70(7), 1067–1081. Available from: 10.2106/00004623-198870070-00016 3042791

[25] Shelburne, K.B. , Torry, M.R. & Pandy, M.G. (2005) Muscle, ligament, and joint‐contact forces at the knee during walking. Medicine & Science in Sports & Exercise, 37(11), 1948–1956. Available from: 10.1249/01.mss.0000180404.86078.ff 16286866

[26] Stoffel, K. , Zderic, I. , Pastor, T. , Woodburn, W. , Castle, R. , Penman, J. et al. (2023) Anterior variable‐angle locked plating versus tension band wiring of simple and complex patella fractures—a biomechanical investigation. BMC Musculoskeletal Disorders, 24(1), 279. Available from: 10.1186/s12891-023-06394-x 37041618 PMC10088273

[27] Thelen, S. , Betsch, M. , Schneppendahl, J. , Grassmann, J. , Hakimi, M. , Eichler, C. et al. (2013) Fixation of multifragmentary patella fractures using a bilateral fixed‐angle plate. Orthopedics, 36(11), e1437–e1443. Available from: 10.3928/01477447-20131021-29 24200450

[28] Torchia, M.E. & Lewallen, D.G. (1996) Open fractures of the patella. Journal of Orthopaedic Trauma, 10(6), 403–409. Available from: 10.1097/00005131-199608000-00007 8854318

[29] Wild, M. , Windolf, J. & Flohé, S. (2010) Patellafrakturen. Der Unfallchirurg, 113(5), 401–412. Available from: 10.1007/s00113-010-1768-x 20446078

[30] Wurm, S. , Bühren, V. & Augat, P. (2018) Treating patella fractures with a locking patella plate—first clinical results. Injury, 49(Suppl 1), S51–S55. Available from: 10.1016/S0020-1383(18)30304-8 29929694

